# Increased Survival in Patients With Molybdenum Cofactor Deficiency Type A Treated With Cyclic Pyranopterin Monophosphate

**DOI:** 10.1002/jimd.70000

**Published:** 2025-03-25

**Authors:** Guenter Schwarz, Donald G. Basel, Bernd C. Schwahn, Ronen Spiegel, Flora Y. Wong, Robin Bliss, Liza Squires

**Affiliations:** ^1^ Department of Chemistry and Biochemistry and Center for Molecular Medicine Cologne University of Cologne Cologne Germany; ^2^ Department of Pediatrics Medical College of Wisconsin Milwaukee Wisconsin USA; ^3^ Manchester Centre for Genomic Medicine, St Mary's Hospital Manchester University NHS Foundation Trust, Health Innovation Manchester Manchester UK; ^4^ Department of Pediatrics B, Emek Medical Center, Afula Rappaport Faculty of Medicine Haifa Israel; ^5^ Department of Pediatrics Monash University; Monash Newborn, Monash Children's Hospital Melbourne Australia; ^6^ Veristat Southborough Massachusetts USA; ^7^ Sentynl Therapeutics Solana Beach California USA

**Keywords:** cPMP, cyclic pyranopterin monophosphate, fosdenopterin, MoCD Type A, molybdenum cofactor deficiency

## Abstract

Molybdenum cofactor deficiency (MoCD) Type A is an ultrarare disorder causing neurodegeneration and early death. Cyclic pyranopterin monophosphate (cPMP), a molybdenum cofactor precursor, is a therapeutic option for patients with MoCD Type A. In this study, efficacy in patients with MoCD Type A treated with recombinant cPMP (rcPMP) and/or fosdenopterin, a synthetic form of cPMP, from one retrospective and two prospective open‐label studies (*N* = 14), was compared with a retrospective/prospective natural history study (untreated; *N* = 37). Safety was evaluated in treated patients. Patients treated with fosdenopterin/rcPMP had significantly reduced risk of premature/early death versus untreated patients (Cox proportional hazards 5.1; 95% CI 1.32–19.36; *p* = 0.01). MoCD disease biomarkers of urinary S‐sulfocysteine and xanthine returned to near‐normal from baseline to last visit in treated patients but remained abnormal in untreated patients. At 12 months, in treated patients, 43% could sit unassisted, 44% were ambulatory, and 57% could feed orally. Initiating fosdenopterin/rcPMP treatment ≤ 14 days after birth appeared to result in better clinical outcomes than initiating > 14 days after birth. Most patients (13/14) had a treatment‐emergent adverse event; most were unrelated to fosdenopterin/rcPMP, were mild to moderate in severity, and none led to treatment discontinuation. These results demonstrate that patients with MoCD Type A who received fosdenopterin/rcPMP versus untreated patients were more likely to survive. Some treated patients were able to feed orally and achieve developmental milestones including walking. Fosdenopterin/rcPMP was generally well‐tolerated. Improved outcomes in patients treated early support the importance of identifying MoCD in neonates and initiating treatment as soon as possible.

## Graphical abstract

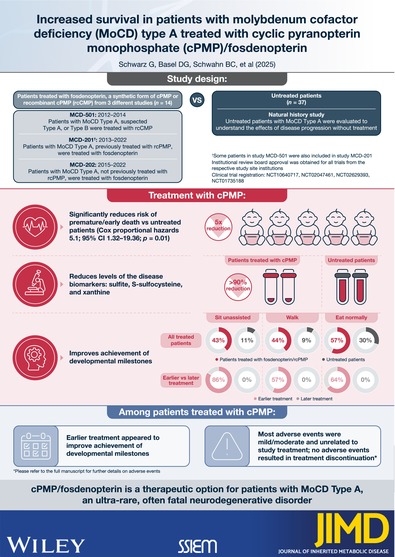

## 1 Introduction

Molybdenum cofactor (MoCo) deficiency (MoCD) is an ultrarare, autosomal recessive, often fatal neurodegenerative inborn error of metabolism, typically presenting in the first hours to days of life [1, 2]. Rapid onset of progressive, irreversible neurologic damage occurs due to loss of function of MoCo‐dependent enzymes, particularly sulfite oxidase [3, 4, 5]. Sulfite oxidase deficiency results in accumulation of highly toxic sulfite and related metabolites, primarily S‐sulfocysteine (SSC) and thiosulfate, accompanied by cystine and homocysteine depletion [6, 7, 8, 9, 10]. MoCD Types A, B, and C, caused by mutations in the *MOCS1*, *MOCS2*, and *GPHN* genes, respectively, are phenotypically indistinguishable in the first weeks of life and require genetic testing for specific diagnosis [3]. Each MoCD type differs based on which enzyme is mutated in the MoCo biosynthesis pathway [3, 5, 11]. MoCD Type A is the most common (≥ 50%) form. In MoCD Type A, the first of the three synthetic steps in the formation of MoCo is interrupted by *MOCS1* mutations preventing formation of cyclic pyranopterin monophosphate (cPMP), resulting in loss of all MoCo‐dependent enzyme activity [12]. Mutations are found in all exons of the *MOCS1* gene encoding for the MOCS1A (exons 1–9) and MOCS1B (exon 10) protein [13]. MoCD Type A worldwide incidence is estimated to be 1 in 200 000 to 500 000 births; however, the exact incidence remains unknown and is likely underdiagnosed [2, 13].

Common presenting symptoms of MoCD Type A in the neonate are intractable seizures, feeding difficulties, encephalopathy, limb hypertonia, and axial hypotonia [3, 10, 14]. Neuroimaging findings on presentation can include cerebral edema, basal ganglia white‐matter changes, and posterior fossa abnormalities on magnetic resonance imaging, which are inconsistent with peripartum history and age [5, 15, 16]. Positive biochemical results from urine or serum and the presence of characteristic symptoms are sufficient to diagnose MoCD. Metabolic findings reflect enzyme deficiencies of sulfite oxidase and xanthine oxidoreductase, including elevated urinary excretion of sulfite, thiosulfate, SSC, xanthine, and hypoxanthine, as well as low cystine, homocysteine, taurine, and uric acid in plasma and urine [17]. Misdiagnoses are common due to similarities with more common causes of neonatal seizures, especially hypoxic‐ischemic injury [17, 18, 19]. At presentation, MoCD Type A cannot clinically be distinguished from other rare diseases, including MoCD Type B or C or isolated sulfite oxidase deficiency. The presentation partly overlaps with other genetic epileptic encephalopathies including pyridoxine‐dependent epilepsy and a correct diagnosis is critical to ensure patients are treated as early as possible, ideally before neurological damage manifests [2].

Untreated patients with MoCD Type A commonly have severe cognitive and motor disabilities and die prematurely. In a retrospective natural history study of 41 untreated patients with MoCD Type A, 1‐year survival rate was 75%, the median survival time was 4.23 years, and the median age at death was 2.4 years [10]. Treatment options for MoCD Type A have historically been supportive, addressing symptoms such as seizures, feeding difficulties, abnormal motor tone, and metabolic acidosis [2, 3]. Although antiepileptic drugs (AEDs) are used, they are typically inadequate for robust seizure control, as chronic seizures secondary to the disease are often refractory to AEDs [20, 21].

Exogenous cPMP is a therapeutic option for patients with MoCD Type A, as cPMP is converted to molybdopterin and then to MoCo [22, 23, 24]. Development of cPMP was established in a *MOCS1*‐deficient mouse model [25] using a recombinant *Escherichia coli* ‐derived, nonsalt, anhydrous form of cPMP (recombinant cPMP; rcPMP) [22, 23, 24, 25, 26]. Further research led to the development of fosdenopterin, a synthetically produced cPMP with identical molecular structure and comparable activity to rcPMP [23, 27]. Preliminary clinical reports suggested that intravenous (IV) administration of rcPMP restored MoCo‐dependent enzyme activities. Normalization of MoCD biomarkers, including urinary SSC levels, was observed [22, 24, 28]. Fosdenopterin was approved by the Food and Drug Administration (FDA) (2021), European Medicines Agency (EMA) (2022), the Israeli Ministry of Health (2022), and the Medicines and Healthcare products Regulatory Agency (MHRA) (2024) for treatment of patients with known or presumed MoCD Type A; however, diagnostic tests for neonatal seizures often do not include MoCD, and MoCD is absent from newborn screening programs. This report presents a long‐term comparison of patients with MoCD Type A from three open‐label studies treated with fosdenopterin/rcPMP (collectively, cPMP), compared with a cohort of untreated patients from a natural history study [10, 22, 24, 28].

## 2 Methods

### 2.1 Study Designs and Participants

**FIGURE 1:**
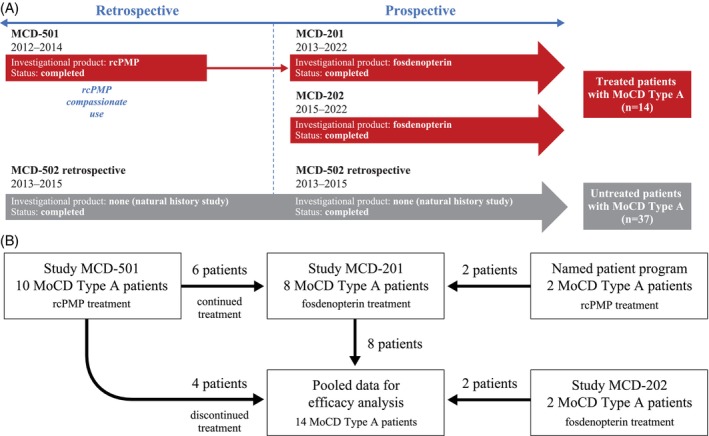
Studies used in comparison of cyclic pyranopterin monophosphate‐treated versus untreated patients with MoCD Type A. (A) Details of treatment studies (MCD‐501, MCD‐201, and MCD‐202) and untreated control study (MCD‐502); and (B) flow chart of treated patients pooled for the full analysis set.

Data evaluating fosdenopterin/rcPMP treatment were derived from three studies (Figure 1A): (1) MCD‐501, a retrospective, observational trial of rcPMP for patients of any age with MoCD Type A, suspected Type A, or Type B; (2) MCD‐201, a prospective, phase II trial of fosdenopterin in patients with MoCD Type A initially treated with rcPMP infusions at time of enrollment; and (3) MCD‐202, a prospective, phase II/III trial of fosdenopterin in neonates, infants, and children with MoCD Type A not previously treated with rcPMP. Data from patients with MoCD Type A from these three trials were pooled in the efficacy full analysis set (FAS; Figure 1B). Institutional review board approval was obtained for all trials from the respective study site institutions.

Fosdenopterin/rcPMP was intravenously administered daily, requiring long‐term indwelling central catheters, and any “device‐related complications” are in reference to central catheters. Dosing of fosdenopterin/rcPMP in the three studies was as follows. Study MCD‐501 treatment was IV rcPMP (free base form) in patients who had previously received rcPMP treatment in accordance with named‐patient treatment plans. Study MCD‐201 treatment was IV fosdenopterin (salt form) starting at 240 μg/kg/day (corresponding to 180 μg/kg/day of the free base form). After 2 months, the dose was escalated monthly by ≤ 240 μg/kg/day until month 6 (corresponding to 180 μg/kg/day of the free base form), until the dose was not tolerable, or until exposure (area under the concentration curve [AUC]) exceeded 5490 μg/kg/day; the maximum dose received by a patient was 1300 μg/kg/day (corresponding to 975 μg/kg/day of the free base form). Study MCD‐202 treatment was IV fosdenopterin (salt form) with dose determined by gestational age (GA): in preterm patients (GA < 37 weeks), day 1 dose was 525 μg/kg/day, while in term patients (GA ≥ 37 weeks), day 1 dose was 700 μg/kg/day. Incremental dose increased at day 28 and month 3 to a maximum of 1200 μg/kg/day, or until the dose was not tolerable or exposure (AUC) exceeded 5490 μg/kg/day. The maximum dose was amended from 1200 to 1300 μg/kg/day (corresponding to 900 to 975 μg/kg/day of the free base form).

Data from untreated controls were from the MCD‐502 trial, a multinational, retrospective and prospective study of patients with MoCD Type A. Data were collected retrospectively for all patients, deceased or living, and prospectively for all living patients who consented to participate.

The efficacy prospective full analysis set (PFAS) comprised patients evaluated prospectively in studies MCD‐502, MCD‐201, and MCD‐202; assessments conducted for each study are provided in the Supporting Information. The efficacy genotype‐matched analysis set (GMAS) included treated patients from the FAS and untreated patients with matching genotypes.

Data from individual studies MCD‐501 (rcPMP‐treated, *n* = 10), MCD‐201 (fosdenopterin‐treated, *n* = 8), and MCD‐202 (fosdenopterin‐treated, *n* = 2) represent the safety population. Six of the patients in study MCD‐501 were also treated in study MCD‐201. Due to the prospective and retrospective nature of the safety information collected, and the variable safety‐reporting specifications required by the different protocols, safety data across studies were not pooled.

### 2.2 Statistical Analysis

The prespecified analysis of overall survival (OS) was conducted using Kaplan–Meier (KM) methods for estimation of survival parameters. The log‐rank test was used to compare OS between treated and untreated patients. In addition to KM methods, OS was analyzed via the Cox proportional hazards model by regressing survival on an indicator variable denoting treatment status. A prespecified subgroup analysis was performed to determine if there was a difference in efficacy among patients who were treated ≤ 14 days of birth versus > 14 days after birth. Descriptive statistics (mean, median, variance, as appropriate) are provided for results that were not included in the prespecified analysis plan.

The Bayley Scales of Infant and Toddler Development (BSID) is a standardized instrument used to evaluate developmental functioning of children aged 1–42 months [29]. Bayley developmental assessment data were administered by trained assessors using the third edition of the BSID. The data are presented graphically using by‐patient spaghetti plots over time for the motor (both gross and fine) and cognitive subtests using age‐equivalent scores and developmental quotient scores. As there were limited retrospective data collected for these assessments, the analysis was conducted only for patients included in the PFAS.

For biomarker analyses, levels of urinary SSC, xanthine, and uric acid (all normalized to urinary creatinine) were analyzed. For the prospective studies, samples were systematically collected at specific timepoints and analyzed by a centralized laboratory using validated assays. For the retrospective studies, biomarkers were collected as available.

Details for methods of data analyses presented in Tables S1–S13 and Figures [Link], [Link] are provided in the Supporting Information.

## 3 Results

### 3.1 Patient Disposition and Characteristics

Overall, data from 51 patients were included in the integrated efficacy analyses, including 14 patients with MoCD Type A who received fosdenopterin/rcPMP (treated patients), and 37 untreated patients with MoCD Type A. As of the data cutoff of October 31, 2020, 9 of 14 (64%) treated patients remained on treatment. Five patients had discontinued treatment, one after 9 days (9 doses of fosdenopterin) per physician's decision, two died at ages 6 days (on treatment) and 15 months (following treatment discontinuation), respectively, and two were reported as off treatment.

Patient demographics were comparable between treated and untreated patients (Table 1). Importantly, however, median age at onset of first MoCD symptom was 1 day (range, 1–5 days) in treated patients and 2 days (range, 1–927 days) in untreated patients. This difference in range highlights that the treated patients had severe neonatal onset, whereas some untreated patients had less severe disease with later onset. The most common presenting signs/symptoms of MoCD Type A in treated versus untreated patients were seizures (71% vs. 92%), feeding difficulties (64% vs. 84%), and high‐pitched cry (50% vs. 43%). A higher proportion of untreated patients had late symptom‐onset (> 28 days after birth) seizures (22%) versus treated patients (7%). Six treated patients were diagnosed in utero, and the age of genetic diagnosis ranged from −181 to 59 days of life. In untreated patients, median age at diagnosis was 269 days (range, 4 days to 40.3 years). Baseline characteristics of the GMAS population were similar to those of the overall population (Table S1).

**TABLE 1:** Baseline characteristics.

Disposition category	cPMP‐treated patients	Untreated controls
	(*n* = 14)	(*n* = 37)
Sex, *n* (%)		
Male	7 (50)	28 (76)
Female	7 (50)	9 (24)
Race, *n* (%)		
White	10 (71)	21 (57)
Asian	4 (29)	10 (27)
Black or African American	0	0
Other	0	6 (16)
Ethnicity, *n* (%)		
Hispanic or Latino	1 (7)	2 (5)
Not Hispanic or Latino	13 (93)	31 (84)
Not reported/unknown	0	4 (11)
Region of birth, *n* (%)		
North America	2 (14)	3 (8)
Europe	6 (43)	14 (38)
Rest of world	6 (43)	20 (54)
Gestational age, median (range), weeks	38.7 (35–41)	39.0 (3641)^a^
Age at genetic diagnosis, median (range), days	3 (−181 to 59)	269 (4–14 708)^a^
Age at onset of first MoCD symptom, median (range), days	1 (1–5)	2 (1–927)
Patients with early seizures, *n* (%)		
No symptoms reported	2 (14)	3 (8)
In utero or during neonatal period	11 (79)	26 (70)
Postneonatal period	1 (7)	8 (22)
MoCD presenting signs and symptoms, *n* (%)		
Seizures	10 (71)	34 (92)
Feeding difficulties	9 (64)	31 (84)
High‐pitched cry	7 (50)	16 (43)
Exaggerated startle response	5 (36)	12 (32)
Metabolic acidosis	4 (29)	7 (19)
Hypertonia	3 (21)	NA
Encephalopathy	3 (21)	NA
Hypotonia	2 (14)	NA
Intracranial hemorrhage	2 (14)	2 (5)
Other	7 (50)	11 (30)

Abbreviations: cPMP, cyclic pyranopterin monophosphate; MoCD, molybdenum cofactor deficiency; NA, not applicable; SD, standard deviation.

^a^ *n* = 30.

### 3.2 Improvement in Survival and in Cognitive and Motor Functioning

**FIGURE 2:**
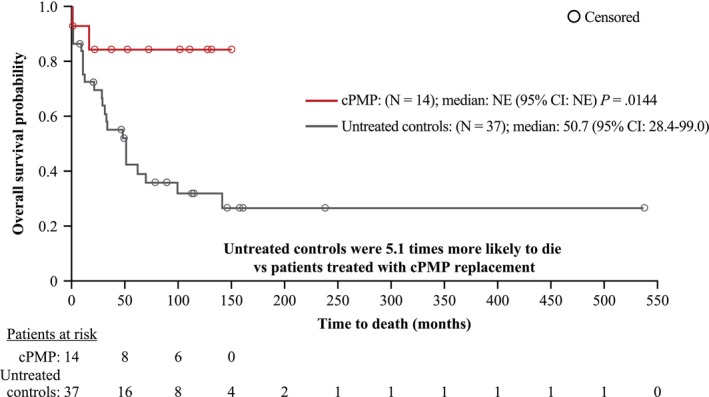
Improvement in survival in patients with MoCD Type A treated with cPMP (FAS, full analysis set).

**FIGURE 3:**
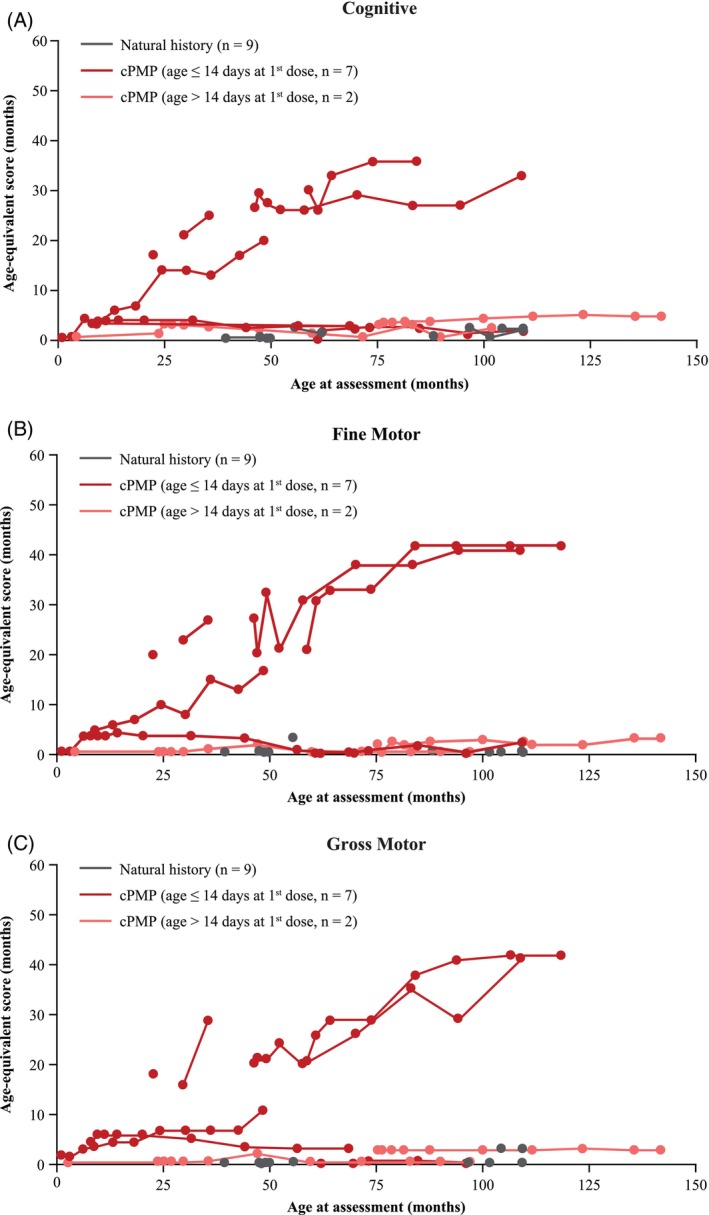
Improvement in cognitive and motor functioning with cPMP treatment. (A) Age‐equivalent scores in months from the Bayley cognitive subtest (prospective FAS); (B) Age‐equivalent scores in months from the Bayley fine motor subtest (prospective FAS); and (C) Age‐equivalent scores in months from the Bayley and gross motor subtest (prospective FAS).

At data cutoff, survival status was confirmed for 38 of 51 patients. Treated patients had statistically significantly prolonged survival versus untreated patients (Figure 2). The risk of death was 5.1 times higher in untreated versus treated patients (Cox proportional hazards 5.1; 95% CI 1.32–19.36; *p* = 0.01). Median OS in treated patients was not reached (95% CI, NE–NE) compared with 50.7 months (95% CI, 28.4–99.0) in untreated patients. Similar OS results were seen in the GMAS (Figure S1). Some patients with MoCD Type A treated with cPMP had higher functioning in developmental outcomes including cognitive (Figure 3A), fine motor (Figure 3B), and gross motor skills (Figure 3C). A review of baseline disease characteristics indicated two distinct patient groups previously treated with cPMP. Four of the eight patients had significant developmental delays consistent with a diagnosis of static encephalopathy at the time of transition from rcPMP to fosdenopterin. This group was non‐ambulatory, microcephalic, had a seizure disorder (on AEDs), and had clinically significant abnormal neuroimaging; three of these four patients were unable to feed by mouth. In contrast, the other four patients walked independently, fed by mouth, and had no history of seizures; these four patients were generally functioning at a developmental level closer to their age level and demonstrated continued developmental progress in the prospective analysis. Based on the review of Bayley and Wechsler Preschool and Primary Scale of Intelligence (WPPSI) age‐equivalent scores, patients who received cPMP were more likely to be higher functioning than the untreated patients.

### 3.3 Urinary Biomarker

Treatment with fosdenopterin/rcPMP led to rapid reduction from baseline in MoCD‐associated urinary biomarkers of SSC (94% reduction) and xanthine (92% reduction), and normal levels of urinary uric acid (all normalized to creatinine; Table 2). Of note, baseline levels of urate in neonates during the first postnatal days reflect a transplacental maternal contribution, whereas with age urate levels drop or are undetectable, as seen in the untreated group (Table 2). Improvements of all biomarkers were maintained over long‐term treatment (range, 30–48 months). In untreated patients, levels of urinary SSC and xanthine remained elevated over time, and urinary uric acid remained low or undetectable. Similar urinary biomarker results were seen in the GMAS (Tables S2–S4).

**TABLE 2:** Mean change in urinary biomarkers: S‐sulfocysteine, xanthine, and uric acid in patients with MoCD Type A treated with cPMP and untreated.

Mean urinary biomarker levels normalized to creatinine; μmol/mmol (SD)	cPMP‐treated patients (*n* = 14)	Untreated controls (*n* = 37)
S‐sulfocysteine		
Baseline	164.2 (263.7), *n* = 14	136.3 (87.2), *n* = 22
Last visit	9.2 (5.6), *n* = 14	156.6 (100.7), *n* = 22
Change to last visit	−155.0 (262.4), *n* = 12	24.8 (104.6), *n* = 18
Xanthine		
Baseline	246.2 (160.9), *n* = 14	315.8 (205.8), *n* = 23
Last visit	19.6 (24.0), *n* = 14	338.2 (233.2), *n* = 23
Change to last visit	−226.6 (168.4), *n* = 14	28.6 (150.7), *n* = 18
Uric acid		
Baseline	444.3 (350.3), *n* = 14	99.1 (165.1), *n* = 20
Last visit	500.6 (219.3), *n* = 14	45.0 (39.3), *n* = 20
Change to last visit	56.4 (484.1), *n* = 14	−67.7 (188.4), *n* = 16

*Note:* Urine reference ranges (0–1 year of age) are 0–18 μmol/mmol creatinine (S‐sulfocysteine), 0–63.4 μmol/mmol creatinine (xanthine), and 820–1026 μmol/mmol creatinine (uric acid) [30].

Abbreviations: cPMP, cyclic pyranopterin monophosphate; MoCD, molybdenum cofactor deficiency; SD, standard deviation.

### 3.4 Developmental Assessments

At the last assessment, 8 of 14 treated (57%) and 10 of 33 untreated (30%) patients were able to feed orally (Figure S2, Table S5). Treated patients had a longer median time before nonoral feeding was required, at 75.0 months, compared with untreated patients, at 10.5 months.

At the last visit, median z‐scores in treated patients were −0.34 for weight, −0.86 for height, and −0.70 for head circumference. In untreated patients, median z‐scores were −0.63 for weight, −1.37 for height, and −1.91 for head circumference (Table S6).

Gross motor function was evaluated using the Gross Motor Function Classification System, Expanded and Revised. At the last assessment, 4 of 9 (44%) treated patients and 1 of 11 (9%) untreated patients were ambulatory (Level I). One treated patient was walking with assistance at 4 years of age (Level III). Most untreated patients (9/11, 82%) required a wheelchair for mobility (Level V).

Nearly half of all treated patients were able to sit unassisted at 12 months of age (43%; 3/7) and at any time (67%; 6/9). In untreated patients, 11% (3/27) were able to sit unassisted at both timepoints (Figure S3).

### 3.5 Seizures and Neurological Examinations

Consistent with MoCD Type A pathophysiology, most patients had seizures that were controlled or ongoing on AED therapy (Table S7). In the prospective data analysis where seizure diaries were collected, at the last assessment in treated and untreated patients: 40% (4/10) and 36% (5/14) had seizures present, 10% (1/10) and 57% (8/14) had seizures controlled, and 50% (5/10) and 7% (1/14) had seizures either not present or resolved (no seizures off AEDs), respectively. There was no statistically significant difference between treated and untreated patients for the likelihood of having seizures not present/resolved versus controlled/continuing.

As expected, based on the MoCD Type A phenotype, most patients in both the treated and untreated groups had abnormal neuroimaging results (Table S8). Furthermore, the majority of patients who completed the neuroimaging assessments experienced no change in findings. One patient from the MCD‐201 trial, who received both rcPMP and fosdenopterin, had an improvement reported from abnormal, clinically significant at the first assessment (3.8 years of age) to abnormal, not clinically significant at 4.4 years of age. Their magnetic resonance imaging (MRI) results continued to be reported as not clinically significant through the last assessment at 6.8 years of age. Among patients in the untreated control group, 33 (89%) of the 37 patients had abnormal results at the first assessment with consistent results reported at the last assessment (35 patients, 95%). Results were similar for the 19 matched control patients in the GMAS with 17 of 19 patients (90%) having abnormal results at the last assessment.

Neurological functioning was evaluated for both groups. Overall, treated patients had fewer abnormal neurological exam findings than untreated patients (Table S9). At the last assessment in treated and untreated patients, abnormal results were observed in 50% (7/14) and 89% (33/37) for truncal tone, 57% (8/14) and 95% (35/37) for appendicular tone, and 64% (9/14) and 81% (30/37) for deep tendon reflexes, respectively.

### 3.6 Treatment Initiation Subgroup Analysis

More than half of the patients initiating treatment ≤ 14 days after birth (*n* = 11) experienced clinically positive outcomes (Table S10). In these patients, 64% (7/11) were able to feed orally, 57% (4/7) were ambulatory, and 86% (6/7) were able to sit unassisted. In patients initiating treatment > 14 days after birth (*n* = 3; at days 32, 37, and 69) none was able to feed orally (0/3), was ambulatory (0/2), or was able to sit unassisted (0/2). Nearly two‐thirds of patients treated ≤ 14 days after birth had seizures resolved, controlled, or never present. There was no apparent difference in OS, and median OS was not estimable in either group. Notably, untreated patients appeared to fare better than patients with treatment initiation > 14 days after birth in the assessments mentioned above (Table S10). This may, in part, be explained by some untreated patients having later onset and less severe disease than treated patients (see Discussion) [10].

### 3.7 Safety

The majority (11/14, 79%) of treated patients were ≤ 14 days of age when they received their first dose of fosdenopterin/rcPMP, including six patients who initiated treatment at 1 day of age; the maximum time to initiate treatment was 69 days. Median overall exposure was 1773 days (4.8 years) and ranged from 6 days to 12.4 years.

Most patients experienced ≥ 1 treatment‐emergent adverse event (TEAE) and ≥ 1 serious adverse event (SAE; Table 3). Most TEAEs were unrelated to study treatment and mild to moderate in severity. In fosdenopterin‐treated patients (*n* = 10 from MCD‐201 and MCD‐202), one patient had two treatment‐related TEAEs (catheter‐site inflammation and central catheter dislocation) and six had a TEAE of severe intensity. In rcPMP‐treated patients (*n* = 10 from MCD‐501), severity and causality were collected for SAEs only, not for every TEAE.

**TABLE 3:** Overall summary of TEAEs (safety set).

TEAE, *n* (%)	MCD‐501^a^ cPMP‐treated (*n* = 10)	MCD‐201 Fosdenopterin‐treated (*n* = 8)	MCD‐202 Fosdenopterin‐treated (*n* = 2)
Any TEAE	9 (90)	8 (100)	2 (100)
Treatment‐related^b^	NA	1 (13)	0
Severe TEAE^b^	NA	4 (50)	2 (100)
Leading to death	2 (20)^c^	0	0
Leading to dose modification	0	0	0
Leading to treatment discontinuation	0	0	0
Most common TEAEs (> 4 patients total)			
Pyrexia	3 (30)	6 (75)	1 (50)
Complication associated with device	0	6 (75)	1 (50)
Pneumonia	3 (30)	3 (38)	1 (50)
Viral infection	0	5 (63)	1 (50)
Otitis media	2 (20)	3 (38)	0
Upper respiratory tract infection	3 (30)	2 (25)	0
Cough	1 (10)	4 (50)	0
Device‐related infection	3 (30)	1 (13)	0
Influenza	0	4 (50)	0
Vomiting	0	3 (38)	1 (50)
Anemia	2 (20)	1 (13)	1 (50)
Any SAE^d^	8 (80)	7 (88)	2 (100)
Treatment‐related SAE	1 (10)	0	0

Abbreviations: NA, not available; SAE, severe adverse event; TEAE, treatment‐emergent adverse event.

^a^ Six of 10 patients in study MCD‐501 were also treated with fosdenopterin in study MCD‐201.

^b^ In study MCD‐501, severity and causality data were collected for SAEs only.

^c^ One patient treated with recombinant cyclic pyranopterin monophosphate (rcPMP) died due to respiratory syncytial virus pneumonia unrelated to study treatment; a second patient treated with rcPMP died due to necrotizing enterocolitis, judged as possibly related to study drug.

^d^ The only SAE to occur in > 1 patient was central venous catheterization (*n* = 2; 1 patient each from MCD‐201 and MCD‐202).

The most common types of TEAEs across studies were catheter‐related complications and infections (Table 3 and Table S11). In rcPMP‐treated patients, the most common TEAEs were pyrexia, pneumonia, upper respiratory tract infection, and device‐related (i.e., long‐term indwelling central catheters) infection (*n* = 3 each). Similarly, the most common TEAEs in fosdenopterin‐treated patients were pyrexia and complications associated with device (*n* = 7 each); viral infection (*n* = 6); pneumonia, cough, vomiting, and influenza (*n* = 4 each); and device dislocation (*n* = 3). There were no TEAEs of phototoxicity across all studies.

The most common SAEs were catheter‐related events and infections (Table S12). One patient had an SAE (necrotizing colitis) considered possibly treatment‐related; all other SAEs were considered unrelated to treatment.

Six patients who received fosdenopterin/rcPMP for suspected MoCD Type A were later confirmed not to have MoCD Type A (MoCD Type B, *n* = 4; unknown type, *n* = 1; unknown diagnosis *n* = 1). These patients received treatment for 3–17 days prior to discontinuation. Two patients experienced TEAEs. With the exception of cardiac failure (*n* = 1) assessed as severe and serious, all other TEAEs were non‐serious and mild or moderate.

Two treated (both rcPMP‐treated) patients with MoCD Type A died; one due to respiratory syncytial virus pneumonia (considered unrelated to study drug by investigator) and the other due to necrotizing enterocolitis (considered possibly related by investigator). One patient with MoCD Type B (rcPMP‐treated) died of unknown cause ~29.6 months after treatment discontinuation.

## 4 Discussion

This study demonstrates that fosdenopterin/rcPMP can reduce the risk of premature/early death in patients with MoCD Type A and is generally well‐tolerated. Treated patients had significantly prolonged survival versus all untreated patients as well as versus genotype‐matched controls. Consistent with its mechanism of action, fosdenopterin/rcPMP treatment led to rapid and sustained reduction of biomarkers of neurotoxic sulfite accumulation as a result of reconstitution of sulfite oxidase activity with newly synthesized MoCo [22, 23, 24].

Considering the rapidity of disease progression, early treatment in patients presenting with clinical and laboratory manifestations of MoCD Type A is imperative, including in patients with suspected, but not confirmed, MoCD Type A. For example, one patient from study MCD‐202 with prenatal diagnosis of MoCD Type A received fosdenopterin within 2 h; by Day 7, his biomarkers were below pathogenic levels and remained below at data cutoff at age 4.3 years. The patient could also feed orally from week 1 to data cutoff and demonstrated continued neurodevelopment. At 4 years of age, he had a cognitive age equivalence of 20 months, could walk independently, and was seizure‐free.

Regrettably, in current clinical practice, most patients with MoCD develop ongoing neurologic damage before accurate diagnosis [3]. Diagnostic tests for neonatal seizures and newborn screening programs often do not include MoCD, and the disease is not included in the Recommended Uniform Screening Panel. In a previous systematic analysis of published MoCD cases, there was a substantial gap between symptom onset and diagnosis [3]. MoCD should be considered in the differential diagnosis of early onset epileptic encephalopathies as it was repeatedly discovered in a cohort of patients suffering from neonatal seizures [10, 31]. Appropriate biochemical and genetic testing should be obtained urgently, with consideration of cPMP replacement therapy for infants with a presumed diagnosis. Additionally, increased prenatal diagnosis of MoCD may also ensure patients with MoCD receive treatment as early as possible. Prenatal monitoring using MRI in fetuses with MoCD Type A has shown possible signs of early brain injury.

Long‐term treatment with fosdenopterin/rcPMP was generally safe in patients with MoCD Type A, as was short‐term treatment in patients without a confirmed Type A diagnosis, providing evidence for viability of treatment before genetic confirmation. No significant safety‐profile differences were observed between rcPMP and fosdenopterin, consistent with the two compounds having identical chemical structures [27]. The most common types of AEs, including SAEs, were related to central‐line complications and to respiratory tract and viral infections. The device‐related complications are consistent with those in patients requiring long‐term indwelling catheters, including device dysfunction, device‐related, and bloodstream infections, and thrombosis [32, 33, 34].

Based in part on the results of these analyses, fosdenopterin was approved by the FDA in February 2021, the EMA in September 2022, the Israeli Ministry of Health in July 2022, and the MHRA in April 2024 for treatment of patients with known or presumed MoCD Type A, and is the first therapy to reduce the risk of premature/early death in these patients. Phototoxic potential was demonstrated with fosdenopterin in standard in vitro and in vivo test systems [35]. During these clinical studies, there were no reports of phototoxicity; however, patients receiving fosdenopterin should minimize or avoid exposure to sunlight [35].

Some limitations should be considered when interpreting these results. Data for the treated group were pooled from three studies with somewhat different populations and dosing strategies. The number of treated patients was relatively low, particularly when divided further to compare ≤and > 14 days of life (as delineated in the prespecified analysis plan); however, this is unavoidable in the field of ultrarare diseases. Additionally, using a historical control limited the study, owing to lack of randomization and blinding, different timing of assessments, and other potentially unknown confounders. In particular, the control group had four patients with postneonatal onset of symptoms and longer survival than most [10]. Thus, we limited formal hypothesis testing to prespecified endpoints that had been discussed with regulatory agencies during development of the statistical analysis plan. Although most patients with MoCD Type A have early onset of symptoms and death, there are atypical cases representing heterogeneity in patients with MoCD. Nonetheless, the multinational, retrospective, and prospective study of patients with MoCD Type A (study MCD‐502) provides the most detailed understanding of the natural history of the disease. The impractical and potentially unethical nature of performing traditional randomized placebo‐controlled trials for ultrarare, deadly diseases often necessitates the use of historical controls, a strategy that has been increasingly used in drug development [36, 37, 38, 39].

Increased awareness and testing for MoCD are needed to ensure patients with MoCD Type A receive life‐saving treatment as rapidly as possible to improve neurological outcome. Treatment with fosdenopterin has the potential to turn MoCD Type A from a neonatal condition that leads to severe disability and is usually fatal, to one with improved survival and neurological outcomes, in particular in those patients that started treatment before manifestation of neurological symptoms. Given its safety profile, early empirical fosdenopterin treatment of encephalopathic infants with potential diagnosis of MoCD Type A should be considered [40, 41].

## Author Contributions

Günter Schwarz, Donald G. Basel, Bernd C. Schwahn, Ronen Spiegel, Flora Wong, and Liza Squires conceptualized and designed the study, drafted the initial manuscript, and critically reviewed and revised the manuscript. Günter Schwarz, Donald G. Basel, Bernd C. Schwahn, Ronen Spiegel, and Flora Wong collected data. Robin Bliss critically reviewed and revised the manuscript. All authors approved the final manuscript as submitted and agreed to be accountable for all aspects of the work.

## Ethics Statement

Institutional review board approval was obtained for all trials from the respective study site institutions.

## Consent

Data were collected retrospectively for all patients, deceased or living, and prospectively for all living patients who consented to participate. All procedures followed were in accordance with the ethical standards of the responsible committee on human experimentation (institutional and national) and with the Helsinki Declaration of 1975, as revised in 2000. Informed consent was obtained from all parents/caregivers of patients for being included in the study.

## Conflicts of Interest

Guenter Schwarz reports conflicts for a member of the advisory board or similar committee with Origin Biosciences, consulting or speaker fee from Sentynl Therapeutics Inc., holding a patent with Sentynl Therapeutics Inc. and founder and CEO of Colbourne Pharmaceuticals GmbH involved in the early stages of development of cPMP therapy. Bernd C. Schwahn reports conflict for an honorarium for the advisory board from Bridge Bio Inc. and an educational grant from Origin Biosciences Inc. Liza Squires reports conflict for consulting or speaker fees from Sentynl Therapeutics Inc. and was employed by Bridge Bio Inc. Donald G. Basel, Ronen Spiegel, Flora Y. Wong, and Robin Bliss have no conflicts of interest to disclose.

## Supporting information


Figure S1.

Figure S2.

Figure S3.

Data S1.

## Data Availability

Deidentified individual participant data will not be made available. Any requests for data by qualified scientific and medical researchers for legitimate research purposes will be subject to review and approval by Sentynl Therapeutics Inc. All requests should be submitted in writing to Sentynl Therapeutics Inc.
