# Supplementary figures and images for: Increased Survival in Patients With Molybdenum Cofactor Deficiency Type A Treated With Cyclic Pyranopterin Monophosphate
Source: J Inherit Metab Dis. 2025 Mar 25;48(2):e70000. doi: 10.1002/jimd.70000 (PMC11936520; doi:10.1002/jimd.70000)

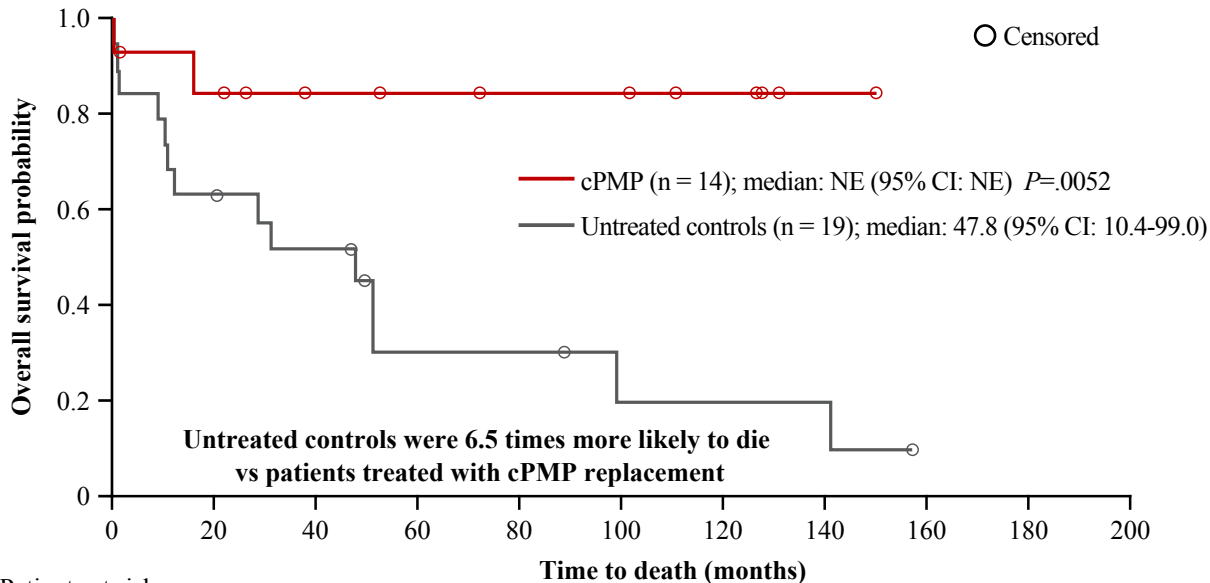

Patients at risk

|                        |    |   |   |   |   |   |   |   |
|------------------------|----|---|---|---|---|---|---|---|
| cPMP: 14               | 10 | 8 | 7 | 6 | 6 | 4 | 1 | 0 |
| Untreated controls: 19 | 12 | 9 | 4 | 4 | 2 | 2 | 2 | 0 |

Supplement: Supplementary file 1 — Figure S1. [file JIMD-48-0-s004.pdf]

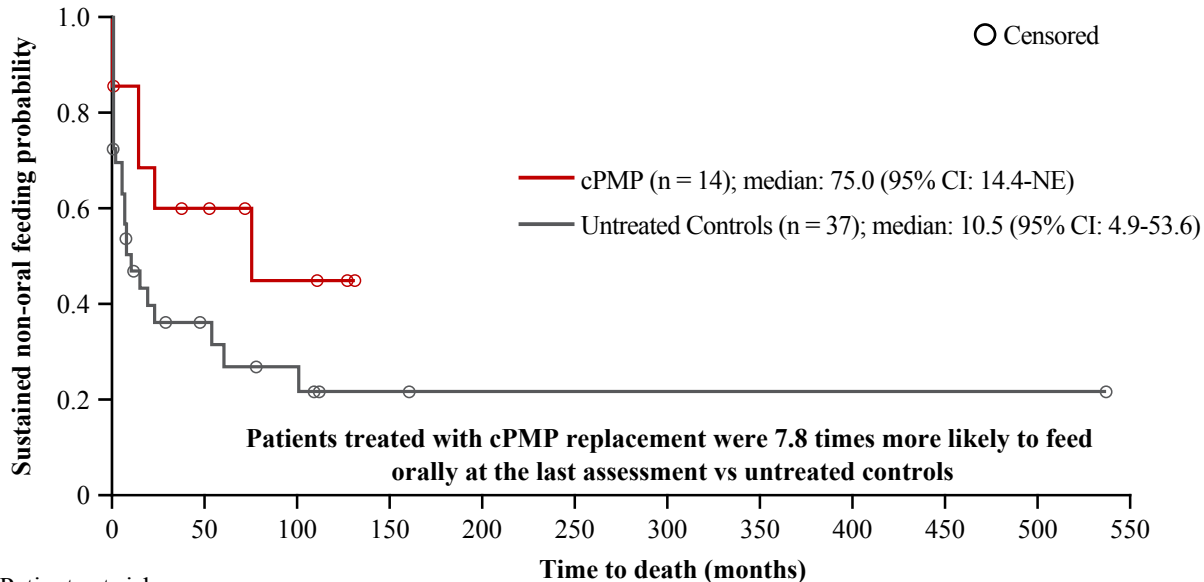

#### Patients at risk

|                        |   |   |   |   |   |   |   |   |   |   |
|------------------------|---|---|---|---|---|---|---|---|---|---|
| cPMP: 13               | 6 | 3 | 0 |   |   |   |   |   |   |   |
| Untreated controls: 33 | 8 | 5 | 2 | 1 | 1 | 1 | 1 | 1 | 1 | 1 |

Supplement: Supplementary file 2 — Figure S2. [file JIMD-48-0-s001.pdf]

**Unassisted sitting by 12 months**

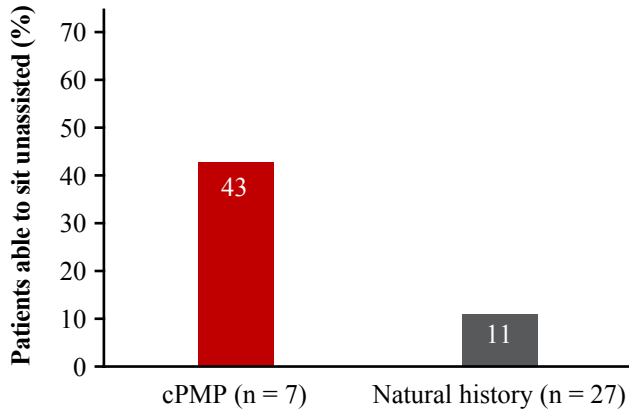

**Unassisted sitting at any time**

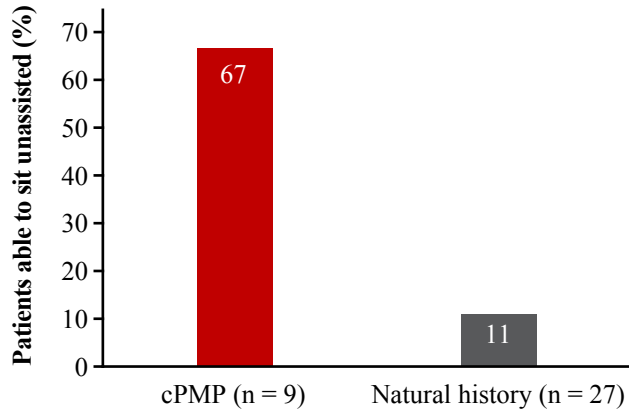

Supplement: Supplementary file 3 — Figure S3. [file JIMD-48-0-s003.pdf]
