# Supplementary material for: Increased Survival in Patients With Molybdenum Cofactor Deficiency Type A Treated With Cyclic Pyranopterin Monophosphate
Source: J Inherit Metab Dis. 2025 Mar 25;48(2):e70000. doi: 10.1002/jimd.70000 (PMC11936520; doi:10.1002/jimd.70000)
Supplement: Supplementary file 4 — Data S1. [file JIMD-48-0-s002.docx]

**Supplemental Information**

**SUPPLEMENTAL METHODS**

Growth parameters (body weight, height, and head circumference) were analyzed by converting each to age- and gender adjusted z-scores. Standard growth curves from the World Health Organization for children up to 5 years of age and growth charts from the Centers for Disease Control and Prevention for children older than 5 years were used.

Feeding patterns were analyzed by frequency and percentages of each feeding method at last visit where feeding pattern was recorded. Time to sustained non-oral feeding was tabulated and plotted using KM methodology, where sustained non-oral feeding was defined as the time at which the patient never subsequently returned to an oral method of feeding.

The GMFCS-ER is a 5-level (Levels I through V) classification system that describes the gross motor function of children and youth (up to 18 years of age) on the basis of their self-initiated movement, with particular emphasis on sitting, walking, and wheeled mobility for children with impaired motor skills. Distinctions among levels are based on functional abilities, the need for assistive technology, including hand-held mobility devices (walkers, crutches, or canes) or wheeled mobility and, to a much lesser extent, quality of movement. The GMFCS-ER classifies gross motor functional capabilities and limitations based on usual performance in the home, school, and community settings. It has been used in a multitude of studies on children with disabilities, including cerebral palsy and Down syndrome. GMFCS-ER data were summarized over time by frequency and percentages at each level of the classification system (Levels I to V). Children who have motor functions classified at “Level I” can generally walk without restrictions, whereas children whose motor function has been classified at “Level V” are very limited in their ability to move themselves around even with the use of assistive technology, and typically are pushed in a wheelchair for their mobility.

The number of patients who were able to sit independently for 30 seconds at 12 months and at any time was tabulated. Only patients who had ≥1 assessment on or after 9 months of age were included in the analysis.

Collection methods of seizure data differed across studies (qualitative vs quantitative depending on the protocol) because of the retrospective chart review and the prospective nature of the studies included. Patients were categorized and tabulated into 4 categories: 1) never had seizures [“not present”], 2) had seizures, but these have resolved without anti-seizure medication [“resolved”], 3) had seizures, which were controlled with anti-seizure medications, defined as no reported seizures in the past 6 months [“controlled”], and 4) still having seizures regularly, defined as >1 seizure in the past 6 months [“present”].

The frequency and percentages of patients with neurological examination findings (spontaneous movement, truncal tone, appendicular tone, deep tendon reflexes, and primitive reflexes) and of normal and abnormal neuroimaging results were assessed over time. Differences between the studies in the reporting of normal and abnormal results were noted; studies MCD-201 and MCD-202 reported results as normal, abnormal clinically significant, and abnormal not clinically significant, whereas in studies MCD-501 and MCD-502, results were reported only as normal or abnormal.

Details of assessments of measures of efficacy across studies are presented in Supplemental Table 13.

SUPPLIMENTAL FIGURE LEGENDS
SUPPLEMENTAL FIGURE 1. Overall Survival in Patients With Molybdenum Cofactor Deficiency Treated With cPMP and Untreated Controls in the Genotype-Matched Analysis Set

Abbreviations: CI, confidence interval; cPMP, cyclic pyranopterin monophosphate; GMAS, genotype-matched analysis set; NE, not evaluable.

**SUPPLEMENTAL FIGURE 2: Time to Sustained Non-oral Feeding for cPMP-Treated Patients and Untreated Controls (Full Analysis Set)**

Abbreviations: CI, confidence interval; cPMP, cyclic pyranopterin monophosphate; NE, not evaluable.

**SUPPLEMENTAL FIGURE 3. Proportion of Patients With the Ability to Sit Unassisted at 12 Months and at Any Time (Full Analysis Set, Patients With Data Available)**

Abbreviations: cPMP, cyclic pyranopterin monophosphate.

SUPPLEMENTAL TABLE 1: Baseline Characteristics Including GMAS

| **Disposition category** | **cPMP-treated patients** (n = 14) | **Untreated controls** | |
| --- | --- | --- | --- |
|  |  | FAS (n = 37) | GMAS (n = 19) |
| **Sex, n (%)** |  |  |  |
| Male | 7 (50) | 28 (76) | 13 (68) |
| Female | 7 (50) | 9 (24) | 6 (32) |
| **Race, n (%)** |  |  |  |
| White | 10 (71) | 21 (57) | 12 (63) |
| Asian | 4 (29) | 10 (27) | 4 (21) |
| Black or African American | 0 | 0 | 0 |
| Other | 0 | 6 (16) | 3 (16) |
| **Ethnicity, n (%)** |  |  |  |
| Hispanic or Latino | 1 (7) | 2 (5) | 0 |
| Not Hispanic or Latino | 13 (93) | 31 (84) | 15 (79) |
| Not reported/unknown | 0 | 4 (11) | 4 (18) |
| **Region of birth, n (%)** |  |  |  |
| North America | 2 (14) | 3 (8) | 0 |
| Europe | 6 (43) | 14 (38) | 9 (47) |
| Rest of world | 6 (43) | 20 (54) | 10 (53) |
| **Gestational age, median (range), weeks** | 38.7 (35, 41) | 39.0 (36, 41)^a^ | 39.0 (37, 40.3)^b^ |
| **Age at genetic diagnosis, median (range), days** | 3 (−181, 59) | 269 (4, 14708)^a^ | 173.5 (4, 1683)^b^ |
| **Age at onset of first MoCD symptom, median (range), days** | 1 (1, 5) | 2 (1, 927) | 2 (1, 222) |
| **Patients with early seizures, n (%)** |  |  |  |
| No symptoms reported | 2 (14) | 3 (8) | 1 (5) |
| In utero or during neonatal period | 11 (79) | 26 (70) | 13 (68) |
| Post-neonatal period | 1 (7) | 8 (22) | 5 (26) |
| **MoCD presenting signs and symptoms, n (%)** |  |  |  |
| Seizures | 10 (71) | 34 (92) | 18 (95) |
| Feeding difficulties | 9 (64) | 31 (84) | 17 (90) |
| High-pitched cry | 7 (50) | 16 (43) | 10 (53) |
| Exaggerated startle response | 5 (36) | 12 (32) | 9 (47) |
| Metabolic acidosis | 4 (29) | 7 (19) | 4 (21) |
| Hypertonia | 3 (21) | NA | NA |
| Hypotonia | 2 (14) | NA | NA |
| Encephalopathy | 3 (21) | NA | NA |
| Intracranial hemorrhage | 2 (14) | 2 (5) | 0 |
| Other | 7 (50) | 11 (30) | 5 (26) |

^a^n = 30. ^b^n = 16.
Abbreviations: cPMP, cyclic pyranopterin monophosphate; FAS, full analysis set; GMAS, genotype-matched analysis set; MoCD, molybdenum cofactor deficiency; NA, not applicable; SD, standard deviation.

SUPPLEMENTAL TABLE 2. Summary of Urinary SSC/Creatinine – First Value, Last Visit, and Change to Last Visit

| **SSC/creatinine (µmol/mmol)** | **cPMP-treated patients** (n = 14) | **Untreated controls** | |
| --- | --- | --- | --- |
|  |  | FAS (n = 37) | GMAS (n = 19) |
| Baseline, first value, n | **14** | **22** | **10** |
| Mean (SD) | 164.2 (263.7) | 136.3 (87.2) | 167.9 (90.5) |
| Median | 84.9 | 114.3 | 156.5 |
| Min, max | 12, 1031 | 2, 345 | 62, 345 |
| Last visit, n | **14** | **22** | **10** |
| Mean (SD) | 9.2 (5.6) | 156.6 (100.7) | 175.0 (102.5) |
| Median | 7.3 | 156.5 | 169.2 |
| Min, max | 4,21 | 11,345 | 11,345 |
| Change to last visit, n | **12** | **18** | **9** |
| Mean (SD) | −155.0 (262.4) | 24.8 (104.6) | 7.9 (102.9) |
| Median | −78.7 | 2.7 | −10.4 |
| Min, max | −1017, −8 | −175, 317 | −175, 153 |

Abbreviations: cPMP, cyclic pyranopterin monophosphate; FAS, full analysis set; GMAS, genotype-matched analysis set; SD, standard deviation; SSC, S-sulfocysteine.

SUPPLEMENTAL TABLE 3. Summary of Urinary Xanthine/Creatinine – First Value, Last Visit, and Change to Last Visit

| **Xanthine/creatinine (µmol/mmol)** | **cPMP-treated patients** (n = 14) | **Untreated controls** | |
| --- | --- | --- | --- |
|  |  | FAS (n = 37) | GMAS (n = 19) |
| Baseline, first value, n | **14** | **23** | **13** |
| Mean (SD) | 246.2 (160.9) | 315.8 (205.8) | 327.3 (194.4) |
| Median | 241.2 | 308.0 | 277.0 |
| Min, max | 26,577 | 0,764 | 0,678 |
| Last visit, n | **14** | **23** | **13** |
| Mean (SD) | 19.6 (24.0) | 338.2 (233.2) | 364.5 (201.6) |
| Median | 14.1 | 277.0 | 338.8 |
| Min, max | 4,100 | 6,937 | 6,678 |
| Change to last visit, n | **14** | **18** | **10** |
| Mean (SD) | −226.6 (168.4) | 28.6 (150.7) | 48.4 (171.8) |
| Median | −220.4 | 6.2 | 9.6 |
| Min, max | −570, −5 | −242, 409 | −242, 409 |

Abbreviations: cPMP, cyclic pyranopterin monophosphate; FAS, full analysis set; GMAS, genotype-matched analysis set; SD, standard deviation.

SUPPLEMENTAL TABLE 4. Summary of Urinary Uric Acid/Creatinine – First Value, Last Visit, and Change to Last Visit

| **Uric acid/creatinine (µmol/mmol)** | **cPMP-treated patients** (n = 14) | **Untreated controls** | |
| --- | --- | --- | --- |
|  |  | FAS (n = 37) | GMAS (n = 19) |
| Baseline, first value, n | **14** | **20** | **12** |
| Mean (SD) | 444.3 (350.3)^a^ | 99.1 (165.11)^a^ | 53.0 (60.00)^a^ |
| Median | 579.7 | 56.7 | 16.0 |
| Min, max | 11, 975 | 7, 750 | 7, 168 |
| Last visit, n | **14** | **20** | **12** |
| Mean (SD) | 500.6 (219.3) | 45.0 (39.3) | 33.7 (43.0) |
| Median | 434.3 | 33.6 | 15.0 |
| Min, max | 224,911 | 4,115 | 4,115 |
| Change to last visit, n | **14** | **16** | **8** |
| Mean (SD) | 56.4 (484.1) | −67.7 (188.4) | −22.9 (67.6) |
| Median | −136.1 | −8.5 | −4.7 |
| Min, max | −517, 900 | −735, 67 | −165, 67 |

^a^Large difference between treated vs untreated uric acid levels may be due to differences in age at recording.
Abbreviations: cPMP, cyclic pyranopterin monophosphate; FAS, full analysis set; GMAS, genotype-matched analysis set; SD, standard deviation.

SUPPLEMENTAL TABLE 5. Analysis of Feeding Status at Last Assessment and Time to Sustained Non-Oral Feeding

| **Feeding status** | **cPMP-treated patients  (n = 14)** | **Untreated controls** | |
| --- | --- | --- | --- |
|  |  | FAS (n = 37) | GMAS (n = 19) |
| **Patients with last feeding assessment, n** | 14 | 33 | 18 |
| **Patients feeding orally, n (%)** | 8 (57) | 10 (30) | 4 (22) |
| **Patients not feeding orally, n (%)** | 6 (43) | 23 (70) | 14 (78) |
| **Logistic regression^a^** |  |  |  |
| Odds ratio (95% CI) |  | 7.8 (1.38-43.84) | 9.1 (1.16-72.39) |
| *P* value |  | 0.020 | 0.036 |
| **Time to non-oral feeding (months)** |  |  |  |
| 75th percentile (95% CI) | NE (75.0-NE) | 100.8 (19.2-NE) | 53.6 (6.5-NE) |
| Median (95% CI) | 75.0 (14.4-NE) | 10.5 (4.9-53.6) | 5.7 (0.2-22.5) |
| 25th percentile (95% CI) | 14.5 (0.0-75.0) | 0.6 (0.1-6.5) | 0.2 (0.1-1.7) |
| Min, Max | 0.0, 75.0 | 0.1, 100.8 | 0.1, 53.6 |

Note: Sustained non-oral feeding is defined as the time at which the patient never subsequently returns to an oral method of feeding.
^a^The logistic regression is fitted using oral feeding (yes/no) as the dependent variable, and treatment status, molybdenum cofactor deficiency symptom onset subgroup, age at last feeding assessment, and sex as independent variables. The odds ratio represents the odds of feeding orally when being treated vs not being treated.
Abbreviations: CI, confidence interval; cPMP, cyclic pyranopterin monophosphate; FAS, full analysis set; GMAS, genotype-matched analysis set; NE, not evaluable.

**SUPPLEMENTAL TABLE 6. Summary of First Value and Last Assessment for Weight, Height, and Head Circumference Z-Scores**

| **Parameter** | **cPMP-treated patients** (n = 14) | **Untreated controls** | |
| --- | --- | --- | --- |
|  |  | FAS (n = 37) | GMAS (n = 19) |
| **Weight z-score at baseline, n** | **14** | **37** | **19** |
| Mean (SD) | −0.18 (0.880) | −0.28 (1.364) | −0.45 (1.538) |
| Median | 0.12 | −0.06 | −0.06 |
| Min, Max | −2.2, 1.4 | −3.7, 2.0 | −3.7, 2.0 |
| **Weight z-score at last visit, n** | **14** | **37** | **19** |
| Mean (SD) | −0.33 (1.237) | −0.70 (1.391) | −0.24 (1.555) |
| Median | −0.34 | −0.63 | −0.25 |
| Min, Max | −2.8, 2.5 | −3.0, 2.8 | −3.0, 2.8 |
| **Height z-score at baseline, n** | **12** | **33** | **16** |
| Mean (SD) | −0.96 (2.724) | −0.44 (2.912) | −0.22 (3.630) |
| Median | −0.14 | 0.18 | 0.25 |
| Min, Max | −8.6, 1.1 | −7.8, 5.4 | −7.8, 5.4 |
| **Height z-score at last visit, n** | **13** | **33** | **16** |
| Mean (SD) | −0.88 (2.394) | −1.05 (2.381) | 0.67 (2.738) |
| Median | −0.86 | −1.37 | −0.80 |
| Min, Max | −7.1, 2.8 | −4.6, 5.4 | −4.4, 5.4 |
| **Head circumference z-score at baseline, n** | **13** | **36** | **19** |
| Mean (SD) | 0.56 (1.121) | −0.79 (2.862) | −1.58 (3.380) |
| Median | 0.52 | 0.07 | −0.32 |
| Min, Max | −1.4, 2.8 | −8.1, 3.5 | −8.1, 3.5 |
| **Head circumference z-score at last visit, n** | **14** | **36** | **19** |
| Mean (SD) | −0.52 (2.393) | −2.03 (2.783) | −2.33 (3.218) |
| Median | −0.70 | −1.91 | −2.95 |
| Min, Max | −5.1, 3.0 | −7.5, 4.3 | −7.5, 4.3 |

Abbreviations: cPMP, cyclic pyranopterin monophosphate; FAS, full analysis set; GMAS, genotype-matched analysis set; SD, standard deviation.

SUPPLEMENTAL TABLE 7. Seizure Status at Last Assessment (Prospective FAS)

| **Disease characteristics, n (%)** | **cPMP-treated patients** (n = 10) | Untreated controls (n = 14) |
| --- | --- | --- |
| **Seizure status at last assessment, n (%)^a^** |  |  |
| Not present | 2 (20) | 1 (7) |
| Resolved | 3 (30) | 0 |
| Controlled | 1 (10) | 8 (57) |
| Present | 4 (40) | 5 (36) |

^a^Seizure status is derived based on the last date of contact. Not Present = Never had seizures; Resolved = Had seizures but have resolved without anti-seizure medication; Controlled = Had seizures and is now controlled with the use of anti-seizure medication, defined as no reported seizures in the past six months; Present = Still having seizures regularly, defined as 1 or more seizures in the last six months.
Abbreviations: cPMP, cyclic pyranopterin monophosphate; FAS, full analysis set.

SUPPLEMENTAL TABLE 8. Summary of Neuroimaging Results (FAS and GMAS)

|  | **cPMP-treated patients** (n = 14) | **Untreated controls** | |
| --- | --- | --- | --- |
|  |  | FAS (n = 37) | GMAS (n = 19) |
| **First value, n (%)** |  |  |  |
| Normal | 1 (8) | 4 (11) | 3 (16) |
| Intermediate | 2 (17) | 0 | 0 |
| Abnormal | 8 (67) | 33 (89) | 16 (84) |
| Abnormal, NCS | 0 | 0 | 0 |
| Abnormal, CS | 1 (8) | 0 | 0 |
| **Last value, n (%)** |  |  |  |
| Normal | 2 (14) | 2 (5) | 2 (11) |
| Intermediate | 0 | 0 | 0 |
| Abnormal | 4 (29) | 35 (95) | 17 (90) |
| Abnormal, NCS | 2 (14) | 0 | 0 |
| Abnormal, CS | 6 (43) | 0 | 0 |

Note that there are differences between the studies with regard to reporting of normal and abnormal results: studies MCD-201 and MCD-202 reported results as 'normal’, 'abnormal not clinically significant', or 'abnormal clinically significant', whereas in studies MCD-501 and MCD-502, results were only reported as 'normal' or 'abnormal’.
Abbreviations: cPMP, cyclic pyranopterin monophosphate; CS, clinically significant; FAS, full analysis set; GMAS, genotype-matched analysis set; NCS, not clinically significant.

SUPPLEMENTAL TABLE 9. Summary of Neurological Examination Results at Last Assessment (FAS and GMAS)

| **Disease characteristics, n (%)** | **cPMP-treated patients** (n = 14)^a^ | **Untreated controls** | |
| --- | --- | --- | --- |
|  |  | FAS (n = 37)^a^ | GMAS (n = 19)^a^ |
| **Spontaneous movement** |  |  |  |
| Normal | 5 (36) | 5 (14) | 2 (11) |
| Abnormal | 8 (57) | 29 (78) | 15 (79) |
| Not examined | 1 (7) | 0 | 0 |
| **Truncal tone** |  |  |  |
| Normal | 3 (21) | 3 (8) | 1 (5) |
| Abnormal | 7 (50) | 33 (89) | 17 (89) |
| **Appendicular tone** |  |  |  |
| Normal | 1 (7) | 1 (3) | 1 (5) |
| Abnormal | 8 (57) | 35 (95) | 17 (89) |
| Not examined | 1 (7) | 0 | 0 |
| **Deep tendon reflexes** |  |  |  |
| Normal | 5 (36) | 3 (8) | 2 (11) |
| Abnormal | 9 (64) | 30 (81) | 15 (79) |
| **Primitive reflexes** |  |  |  |
| Normal | 1 (7) | 0 | 0 |
| Abnormal | 2 (14) | 0 | 0 |
| Not examined | 2 (14) | 0 | 0 |

N-values do not always add up to total because results are for data collected prospectively.
Abbreviations: cPMP, cyclic pyranopterin monophosphate; FAS, full analysis set; GMAS, genotype-matched analysis set.

SUPPLEMENTAL TABLE 10. Select Outcomes in Patients Treated Early (≤14 Days Of Birth) Vs Late (>14 Days After Birth) and Untreated Patients

| **Assessment, n/N (%)** | **cPMP-treated patients** | | **Untreated patients  (n = 37)** |
| --- | --- | --- | --- |
|  | Treatment initiation ≤14 days  (n = 11) | Treatment initiation >14 days  (n = 3) |  |
| **Oral feeding at last assessment** | 7/11 (64) | 0/3 (0) | 10/33 (30) |
| **Ambulatory (GMFCS-1)** | 4/7 (57) | 0/2 (0) | 1/11 (9) |
| **Sitting unassisted at any time** | 6/7 (86) | 0/2 (0) | 3/27 (11) |
| **Seizures resolved, controlled, or never present** | 7/11 (64) | 0/3 (0) | 24/37 (65) |

Abbreviations: cPMP, cyclic pyranopterin monophosphate; GMFCS-1, Gross Motor Function Classification System Level 1.

SUPPLEMENTAL TABLE 11. Treatment-Emergent Adverse Events Reported in >1 Patient (Safety Set, Patients With MoCD Type A)

| System organ class, preferred term, n (%) | MCD-501 (n = 10) | MCD-201 (n = 8) | MCD-202 (n = 2) |
| --- | --- | --- | --- |
| **Patients with ≥1 AE** | **9 (90)** | **8 (100)** | **2 (100)** |
| **Infections and infestations** | **8 (80)** | **8 (100)** | **1 (50)** |
| Pneumonia | 3 (30) | 3 (38) | 1 (50) |
| Viral infection | 0 | 5 (63) | 1 (50) |
| Otitis media | 2 (20) | 3 (38) | 0 |
| Upper respiratory tract infection | 3 (30) | 2 (25) | 0 |
| Device-related infection | 3 (30) | 1 (13) | 0 |
| Influenza | 0 | 4 (50) | 0 |
| Sepsis | 2 (20) | 2 (25) | 0 |
| Catheter-site infection | 0 | 2 (25) | 1 (50) |
| Gastroenteritis | 1 (10) | 1 (13) | 1 (50) |
| Gastroenteritis: viral | 0 | 2 (25) | 1 (50) |
| Oral candidiasis | 2 (20) | 1 (13) | 0 |
| Varicella | 2 (20) | 1 (13) | 0 |
| Bacteremia | 0 | 1 (13) | 1 (50) |
| Bronchitis | 1 (10) | 1 (13) | 0 |
| Device-related sepsis | 2 (20) | 0 | 0 |
| Ear infection | 0 | 2 (25) | 0 |
| Fungal skin infection | 2 (20) | 0 | 0 |
| Lower respiratory tract infection | 0 | 2 (25) | 0 |
| Nasopharyngitis | 0 | 2 (25) | 0 |
| Otitis media: acute | 0 | 1 (13) | 1 (50) |
| Respiratory tract infection | 1 (10) | 1 (13) | 0 |
| Urinary tract infection | 1 (10) | 1 (13) | 0 |
| Vascular device infection | 0 | 2 (25) | 0 |
| Viral tonsillitis | 0 | 1 (13) | 1 (50) |
| Viral upper respiratory tract infection | 0 | 2 (25) | 0 |
| **General disorders and administration site conditions** | **8 (80)** | **7 (88)** | **1 (50)** |
| Pyrexia | 3 (30) | 6 (75) | 1 (50) |
| Complication associated with device | 0 | 6 (75) | 1 (50) |
| Catheter-site discharge | 0 | 2 (25) | 0 |
| Catheter-site extravasation | 0 | 2 (25) | 0 |
| Catheter-site inflammation | 1 (10) | 1 (13) | 0 |
| Catheter-site pain | 0 | 2 (25) | 0 |
| Device dislocation^a^ | 2 (20) | 0 | 0 |
| Device leakage^a^ | 2 (20) | 0 | 0 |
| Medical device complication | 2 (20) | 0 | 0 |
| **Respiratory, thoracic, and mediastinal disorders** | **5 (50)** | **7 (88)** | **1 (50)** |
| Cough | 1 (10) | 4 (50) | 0 |
| Sneezing | 1 (10) | 2 (25) | 0 |
| Asthma | 1 (10) | 1 (13) | 0 |
| Epistaxis | 0 | 2 (25) | 0 |
| Oropharyngeal pain | 0 | 2 (25) | 0 |
| Rhinorrhea | 1 (10) | 0 | 1 (50) |
| **Skin and subcutaneous tissue disorders** | **5 (50)** | **7 (88)** | **1 (50)** |
| Rash | 0 | 3 (38) | 0 |
| Dermatitis | 1 (10) | 0 | 1 (50) |
| Eczema | 2 (20) | 0 | 0 |
| Rash: maculo-papular | 0 | 2 (25) | 0 |
| Skin disorder | 0 | 2 (25) | 0 |
| **Gastrointestinal disorders** | **4 (40)** | **6 (75)** | **1 (50)** |
| Vomiting | 0 | 3 (38) | 1 (50) |
| Diarrhea | 0 | 2 (25) | 1 (50) |
| Abdominal pain | 0 | 2 (25) | 0 |
| Constipation | 1 (10) | 1 (13) | 0 |
| **Injury, poisoning, and procedural complications** | **0** | **6 (75)** | **1 (50)** |
| Contusion | 0 | 1 (13) | 1 (50) |
| **Blood and lymphatic system disorders** | **2 (20)** | **3 (38)** | **1 (50)** |
| Anemia | 2 (20) | 1 (13) | 1 (50) |
| Eye disorders | 2 (20) | 3 (38) | 1 (50) |
| Conjunctival hemorrhage | 1 (10) | 0 | 1 (50) |
| Eye swelling | 0 | 2 (25) | 0 |
| Strabismus | 1 (10) | 1 (13) | 0 |
| **Nervous system disorders** | **1 (10)** | **4 (50)** | **0** |
| Seizure | 0 | 2 (25) | 0 |
| **Metabolism and nutrition disorders** | **0** | **2 (25)** | **2 (100)** |
| Hypoglycemia | 0 | 0 | 2 (100) |
| Product issues | 0 | 4 (50) | 0 |
| Device dislocation^a^ | 0 | 3 (38) | 0 |
| Device leakage^a^ | 0 | 2 (25) | 0 |
| Device occlusion | 0 | 2 (25) | 0 |
| **Psychiatric disorders** | **1 (10)** | **3 (38)** | **0** |
| Agitation | 0 | 2 (25) | 0 |
| Irritability | 1 (10) | 1 (13) | 0 |
| Surgical and medical procedures | 2 (20) | 1 (13) | 1 (50) |
| Central venous catheterization | 0 | 1 (13) | 1 (50) |

Note: Six of the 10 patients in study MCD-501 were also treated with fosdenopterin in study MCD-201. ^a^Coding was conducted using MedDRA version 17.0 in study MCD-501 and MedDRA version 21.1 in studies MCD-201 and MCD-202; the System Organ Class for these preferred terms (device dislocation and device leakage) was modified between these 2 versions of the dictionary.
Abbreviations: AE, adverse event.

SUPPLEMENTAL TABLE 12. Serious Treatment-Emergent Adverse Events Reported in >1 Patient (Safety Set)

| **System organ class, preferred term, n (%)** | **MCD-501 (n = 10)** | **MCD-201 (n = 8)** | **MCD-202 (n = 2)** |
| --- | --- | --- | --- |
| **Subjects with ≥1 SAE** | **8 (80)** | **7 (87.5)** | **2 (100)** |
| **Infections and infestations** | **6 (60)** | **6 (75)** | **1 (50)** |
| Pneumonia | 2 (20) | 2 (25) | 1 (50) |
| Device-related infection | 3 (30) | 1 (12.5) | 0 |
| Sepsis | 2 (20) | 1 (12.5) | 0 |
| Bacteremia | 0 | 1 (12.5) | 1 (50) |
| Catheter-site infection | 0 | 2 (25) | 0 |
| Device-related sepsis | 2 (20) | 0 | 0 |
| Lower respiratory tract infection | 0 | 2 (25) | 0 |
| Vascular device infection | 0 | 2 (25) | 0 |
| Viral infection | 0 | 1 (12.5) | 1 (50) |
| **General disorders and administration site conditions** | **5 (50)** | **5 (62.5)** | **1 (50)** |
| Complication associated with device | 0 | 4 (50) | 1 (50) |
| Pyrexia | 2 (20) | 2 (25) | 0 |
| Medical device complication | 2 (20) | 0 | 0 |
| Device dislocation^a^ | 2 (20) | 0 | 0 |

^a^Coding was conducted using MedDRA version 17.0 in study MCD-501 and MedDRA version 21.1 in studies MCD-201 and MCD-202; the System Organ Class for these preferred terms (device dislocation and device leakage) was modified between these 2 versions of the dictionary.
Abbreviations: SAE, serious adverse event.

SUPPLEMENTAL TABLE 13. Assessment of Measures of Efficacy Across Studies

| Study: | MCD-502^a^ Natural History | | MCD-501^a^ | MCD-201 | MCD-202 |
| --- | --- | --- | --- | --- | --- |
| Treatment: | None | | rcPMP | Fosdenopterin | Fosdenopterin |
| Data collection: | Retrospective | Prospective | Retrospective | Prospective | Prospective |
| **Biomarkers** | | | | | |
| Urine biomarkers | SSC, UA, xanthine, creatinine | SSC, UA, xanthine, creatinine | SSC, UA, xanthine, creatinine | SSC, UA, xanthine, creatinine, urothione | SSC, UA, xanthine, creatinine, urothione |
| Assessments conducted | Records collected as available | At enrollment, weekly from birth to 1 month of age, monthly until 3 months of age, and then every 3 months | Records collected as available | Screen/BL; Days 1, 4^b^, 7, 14, 28, 57, 67, 87, 97, 117, 127, 147, 157, 180; Months: 9, 12, 18, 24, 30, 36, 48, 60, 78, every 12 months thereafter; Safety FUP; first day of dose adjustment and 7-day FUP following dose adjustment | Screen/BL; Days 1, 2b, 3b, 4, 5b, 6b, 7, 14, 28, 56; Months 3, 4b, 5b, 6, 9, 12, 18, 24, 30, 36; Safety FUP/ET; 1st day of dose adjustment and 7-day FUP following dose adjustment |
| **Developmental assessments** | | | | | |
| GMFCS-ER | Records collected as available | BL and at Months 6 and 12 as available | Records collected as available | Screen; Days 28, 90, 180; Months 12, 24, 36, 48, 60, 66, 78, and every 12 months thereafter; Safety FUP | Months 12, 24, 36, and Safety FUP/ET |
| Bayley | Records collected as available | At 3 months of age, and every 6 months as available | Records collected as available | BL; Day 28; Months 3, 6, 12, 24, 36, 48, 60, 66, 78, and every 12 months thereafter | Day 28; Months 3, 6, 9, 12, 18, 24, 30, 36; Safety FUP/ET |
| WPPSI | Records collected as available | At 3 years of age and at the end of the 1-year prospective evaluation as available | Records collected as available | Screen; Months 6, 12, 24, 36, 48, 60, 66, 78, every 12 months thereafter, and when appropriate | Day 28; Months 3, 6, 9, 12, 18, 24, 30, 36; Safety FUP/ET, if applicable |
| Denver | Records collected as available | BL and every 3 months thereafter as available | Records collected as available | Not assessed | Not assessed |
| GMFM-88 | Not assessed | Not assessed | Not assessed | Not assessed | Day 28; Months: 3, 6, 9, 12, 18, 24, 30, 36; Safety FUP/ET |
| Ability to sit unassisted | As measured by the Denver Developmental Screening Test:  Sit – No Support^c^.  As measured by Bayley Item #26: Sits without support for 30 seconds.  Neurologic exam includes the following question: Is the patient able to sit without support for 30 seconds or longer and at what age did the patient achieve this milestone? | As measured by the Denver Developmental Screening Test:  Sit – No Support^c^  As measured by Bayley Item #26: Sits without support for 30 seconds | As measured by the Denver Developmental Screening Test:  Sit – No Support^c^.  As measured by Bayley: item #26: Sits without support for 30 seconds | As measured by Bayley Item #26: Sits without support for 30 seconds | As measured by Bayley Item #26: Sits without support for 30 seconds  As measured by the GMFM-88: Item 24: Sitting on mat: Maintains, arms free, 3 seconds |
| PEDI | Not assessed | Not assessed | Not assessed | Not assessed | Months 6, 12, 24, 36; Safety FUP/ET |
| **Neuroimaging** | | | | | |
| Types of neuroimaging | MRI  CT scan  Ultrasound | MRI  CT scan  Ultrasound | MRI  CT scan  Ultrasound | MRI  CT scan | MRI  Ultrasound |
| Results collected: | Normal  Abnormal | Normal  Abnormal | Normal  Abnormal  Indeterminate | Normal  Abnormal  Indeterminate | Normal  Abnormal Not clinically significant  Clinically significant |
| Assessment conducted: | Records collected as available | BL, Months 6 and 12 (if clinical condition allowed) | Records collected as available | Screen/BL; Months 6, 12, 24, 36, 60, 66, 78, and every 12 months thereafter.  Neuroimaging is optional if the patient’s clinical status has not changed since the Month 6 assessment | MRI  Screen/BL; Months 24, 36, and if clinical conditions allow, additional scans may be requested if clinically indicated.  Ultrasound  Screen/BL, Day 3, 21 (neonates only) |
| **Seizure activity:** | | | | | |
| Seizure type captured? | Yes | Yes | Yes | No | No |
| Seizure counts collected? | No | Yes | Yes | Yes | Yes |
| Collection method: | Chart review | Daily diary | Chart review | Daily diary | Daily diary |
| Anti-epileptic drugs? | General question on seizure CRF plus Con med page | General question on seizure CRF plus Con med page | Captured on specific CRF | General question on seizure CRF plus Con med page | General question on seizure CRF plus Con med page |
| Assessments conducted: | Retrospective collection from birth to time of enrollment | Assessed continuously during 12-month observation period | Retrospective data collection included all available data with suggested time points as follows: BL; Days 1–14; Months 1, 2, 3, and every 3 months | Screen; Days 7, 14, 28, then monthly | Screen; Days 7, 14, 21, 28, then monthly; 1st day of dose adjustment and  7-day FUP following dose adjustment |
| **Neurological examinations:** | | | | | |
| Parameters examined | Spontaneous movement, truncal tone, appendicular tone, deep tendon reflexes, primitive reflexes, dystonic, opisthotonic, clonus, ambulation, Communication | Spontaneous movement, truncal tone, appendicular tone, deep tendon reflexes, primitive reflexes, dystonic, opisthotonic, clonus, ambulation, communication | Spontaneous movement, truncal tone, appendicular tone, deep tendon reflexes, primitive reflexes | Spontaneous movement, truncal tone, appendicular tone, deep tendon reflexes, primitive reflexes, dystonic, opisthotonic, clonus, ambulation, communication | Spontaneous movement, truncal tone, appendicular tone, deep tendon reflexes, primitive reflexes, dystonic, opisthotonic, clonus |
| Assessments conducted: | Retrospective data collection included all data from birth to 1 month of age. Data from 1 month to time of enrollment collected at intervals no shorter than 1 month | At enrollment and then weekly from birth to 1 month of age, monthly until 3 months of age, and then every 3 months | Retrospective data collection included all available data with suggested time points as follows: BL; Days 7, 14; Months 1, 2, 3, and then every 3 months | Screen/BL; Days 1, 4, 7, 14, 28, 60, 90, 120, 150, 180; Months 9, 12, 18, 24, 30, 36, 42 48, 54, 60, 66, 72 and every 6 months thereafter, 78 and every 12 months thereafter; Safety FUP; first day of any dose adjustment and 7-day FUP following any unscheduled dose adjustment | Screen/BL; Days 1, 4, 7, 14, 28; Months: 3, 4, 5, 6, 9, 12, 18, 24, 30, 36; Safety FUP/ET; first day of any dose adjustment and 7-day FUP following dose adjustment |

^a^Study MCD-502 also collected available data on homocysteine, methionine, taurine, hypoxanthine, sulfite, and thiosulfate in urine and homocysteine, methionine, taurine, and hypoxanthine in plasma. Study MCD-501 also collected available data on sulfite and thiosulfate in urine. ^b^Assessments on these days were conducted in urine only.
^c^The Denver Developmental Screening test does not specify for 30 seconds.
Abbreviations: BL, baseline; Bayley, The Bayley Scales of Infant Development, Third Edition; Con, concomitant; CRF, case report form; CT, computerized tomography; ET, end of treatment; FUP, follow-up; GMFCS-ER, Gross Motor Function Classification System, Expanded and Revised; GMFM-88, Gross Motor Function Measure-88; med, medication; MRI, magnetic resonance imaging; PEDI, Pediatric Evaluation of Disability Inventory; rcPMP, recombinant sourced cyclic pyranopterin monophosphate; Screen, screening; SSC, S-sulfocysteine; UA, uric acid; WPPSI, Wechsler Preschool and Primary Scale of Intelligence.
